# CCl_4_-Induced Liver Injury Was Ameliorated by Qi-Ge Decoction through the Antioxidant Pathway

**DOI:** 10.1155/2019/5941263

**Published:** 2019-12-31

**Authors:** Chong Peng, Zun-ming Zhou, Jing Li, Yan Luo, Yun-song Zhou, Xue-hong Ke, Ke-er Huang

**Affiliations:** ^1^The First Clinical Medical School, Guangzhou University of Chinese Medicine, Guangzhou 510405, Guangdong, China; ^2^Lingnan Medical Research Center, Guangzhou University of Chinese Medicine, Guangzhou 510405, Guangdong, China

## Abstract

Qi-Ge decoction (QGD), which is derived from the Huangqi Gegen decoction, contains three traditional Chinese herbs: *Astragalus membranaceus* (Huangqi), *Pueraria lobata* (Gegen), and *Citri Reticulatae Blanco Pericarpium* (Chenpi). Gastric mucosal damage caused by ethanol was prevented and alleviated by QGD. However, the role of QGD in protecting the liver from toxins has not been reported. High-performance liquid chromatography with diode-array detection was used to qualitatively analyze QGD. Positive control (silymarin 100 mg/kg/day), QGD (20, 10, or 5 g/kg/day), and Nrf2 inhibitor brusatol (0.4 mg/kg/2 d) were administered to rats for 7 days, and then, liver injury was induced by injecting 2 mL/kg 25% CCl_4_. After 24 h, blood and liver were collected for analysis and evaluation. QGD was found to contain 12 main components including calycosin, puerarin, and hesperidin. QGD treatment significantly reduced liver damage and decreased serum alanine aminotransferase, aspartate aminotransferase, and lactate dehydrogenase activities. QGD increased superoxide dismutase and catalase activities, and glutathione levels, but decreased malondialdehyde levels in livers from CCl_4_-treated rats. Compared to rats treated with CCl_4_ alone, after QGD administration, mRNA and protein levels of nuclear factor erythroid 2-related factor 2 (Nrf2) and heme oxygenase-1 were increased, while those of Kelch-like ECH-related protein 1 (Keap1) and cytochrome P450 (CYP)2E1 were decreased. However, these improvements in QGD were reversed by brusatol. In conclusion, QGD can achieve its hepatoprotective effect through an antioxidant mechanism by activating the Nrf2 pathway.

## 1. Introduction

An important role of the liver is detoxification [1]. Dysfunction of detoxification and the metabolic pathways of the liver lead to liver injury and various liver diseases such as hepatitis, fatty liver disease, and fibrosis [2–4]. Currently, drugs used to treat liver injury are limited in efficacy or elicit many adverse reactions [5, 6].

The drug pair *Astragalus membranaceus* (Huangqi)-*Pueraria lobata* (Gegen) was originally described in “Zhengzhihuibu,” written during the Qing Dynasty of China. Many Chinese doctors believe that this drug pair exerts important detoxification and hepatoprotective effects [7]. Under the guidance of traditional Chinese medicine theory, Qi-Ge decoction (QGD), consisting of the Huangqi Gegen drug pair and *Citri Reticulatae Blanco Pericarpium* (Chenpi), has been used as a clinically effective formula for treating liver damage diseases for many years in China [8, 9]. In addition, a previous study has shown that QGD exerted hepatoprotective effects by reducing serum ALT and AST levels in a liver injury mice model [10].

A recent study has shown that *Astragalus membranaceus* protects tissues such as the brain, kidney, intestine, and liver from oxidative stress, and its antioxidant function is achieved by scavenging reactive oxygen species (ROS) and free radicals [11]. In addition, acetaminophen-induced liver damage can be alleviated by *Astragalus membranaceus* by activating the nuclear factor erythroid 2-related factor 2 (Nrf2) antioxidant stress pathway, reducing the expression of Kelch-like ECH-related protein 1 (Keap1), and increasing the expression of heme oxygenase-1 (HO-1) [12]. A pharmacological study has also shown that Pueraria protects the liver and nervous system from injury by inhibiting oxidation and apoptosis both in vivo and in vitro [13]. However, studies investigating the mechanisms of QGD to protect against liver injury have not been reported.

In this study, the protective effects of QGD on the liver were evaluated in a classical model of CCl_4_-induced acute liver injury in rats. Furthermore, the potential antioxidant mechanisms of QGD were explored.

## 2. Materials and Methods

### 2.1. Reagents and Drugs

Huangqi (*Astragalus membranaceus*) and Chenpi (*Citri Reticulatae Blanco Pericarpium*) were purchased from the First Affiliated Hospital of Guangzhou University of Chinese Medicine (Guangzhou, China). Gegen (*Pueraria lobata*) was purchased from Beijing Tongrentang Anhui (Bozhou) Pieces (Beijing, China). Silymarin was purchased from Guangzhou Ruishu Biological Technology (Guangzhou, China). CCl_4_ was purchased from Guangzhou Chunchang Biotechnology Development (Guangzhou, China). Aspartate aminotransferase (AST), alanine aminotransferase (ALT), and lactate dehydrogenase (LDH) assay kits were purchased from Beijing Jiuqiang Biotechnology (Beijing, China). Superoxide dismutase (SOD), malondialdehyde (MDA), reduced glutathione (GSH), and catalase (CAT) assay kits were purchased from Nanjing Jiancheng Bioengineering Institute (Jiangsu, China). Nrf2, Keap1, cytochrome P450 (CYP) 2E1, and HO-1 antibodies were purchased from Proteintech (Rosemont, IL, USA). Brusatol was purchased from Sigma (Sigma-Aldrich, St. Louis, MO, USA).

### 2.2. Preparation of QGD

The QGD preparation (shown in Table 1) contains three traditional Chinese herbs: *Astragalus membranaceus* (180 g), *Pueraria lobata* (60 g), and *Citri Reticulatae Blanco Pericarpium* (30 g) (dry weight ratio 6 : 2 : 1, respectively) mixed in 5400 mL distilled water (1/20, w/v). These herbs were authenticated by Prof. Xiao-Hong Yuan (Guangzhou University of Chinese Medicine, China). The first decoction portion was obtained after the first boil and simmer for 30 min, and the other two portions were obtained after adding 4050 mL of distilled water (1/15, w/v) and boiling and simmering twice for 30 min each time. The three decoction portions were combined, mixed, and concentrated to crude drug of 1 g/mL of drug solution.

### 2.3. Sample Processing and High-Performance Liquid Chromatography with Diode-Array Detection (HPLC-DAD) Analysis

QGD (0.5 mL) was added to 9.5 mL of 50% methanol for ultrasonic extraction for 30 min. It was then cooled at room temperature, and 50% methanol was added to compensate for volume loss. The decoction was passed through a 0.22 *μ*m filter prior to use.

Fingerprinting of QGD was performed on a Hypersil ODS2 C18 column (4.6 × 250 mm, 5 *μ*m pore size; Elite, Dalian, China) in a Waters 2695 series HPLC-DAD. The mobile phase consisted of water containing 0.1% citric acid (A) and methanol (B). The solvent gradient elution parameters were 0 min, 10% B; 20 min, 25% B; 45 min, 35% B; 50 min, 37% B; 55 min, 37% B; 60 min, 45% B; 65 min, 50% B; 70 min, 60% B; 75 min, 70% B; 80 min, 80% B; and 85 min, 90% B. The column temperature was 30°C, the flow rate was 1.0 mL/min, and the injection volume was 20 *µ*L. All standards and samples were detected by absorbance at 250 nm. The standard chemicals used were calycosin-7-glucoside, daidzein, daidzin, formononetin, genistein, genistin, hesperidin, and puerarin (National Institutes for Food and Drug Control, China); 3′-hydroxy puerarin, nobiletin, and tangeretin (Cato Corporation, Canada); and calycosin (Sigma-Aldrich, St. Louis, MO, USA). Retention times of the various compounds were determined under the same analytical conditions and compared to the UV spectra of the DAD library to determine the composition of QGD.

### 2.4. Animals and Treatments

Sixty male Sprague-Dawley rats weighing 200–220 g were used in the study. All animals were purchased from the Experimental Animal Center of Guangzhou University of Chinese Medicine. Animal procedures were approved by the Ethics Committee of Guangzhou University of Chinese Medicine. Rats were maintained in a 12 : 12 h light/dark cycle, temperature of 22 ± 2°C, and relative humidity of 65 ± 5%, and allowed free access to food and water.

1. Rats were divided into 6 groups including control (saline), CCl_4_ + saline, CCl_4_ + positive control (silymarin 100 mg/kg/day), and CCl_4_ + QGD (20, 10, or 5 g/kg/day). There were ten rats in each group. QGD was administered orally at doses of 20, 10, or 5 g/kg. Silymarin was administered orally at 100 mg/kg. Saline solution was administered orally to the control and CCl_4_ alone groups.
2. In another experiment, rats were divided into 4 groups including CCl_4_, CCl_4_ + brusatol (brusatol 0.4 mg/kg/2 d, ip) [14], CCl_4_ + QGD (20 g/kg/day), and CCl_4_ + brusatol + QGD.

After seven days of treatment, the control group was injected intraperitoneally with paraffin oil and the other groups with a 25% CCl_4_-paraffin oil mixture (2 mL/kg body weight). Twenty-four hours after treatment, all rats were anesthetized by an intraperitoneal injection of sodium pentobarbital (30 mg/kg) and sacrificed. Blood was collected from the abdominal aorta and centrifuged at 3000 *g* before biochemical analysis. The liver was immediately removed and stored at −80°C or fixed in 10% paraformaldehyde.

### 2.5. Measurements of Serum Enzymes and Liver Components and Enzymes

Serum ALT, AST, and LDH activities were measured by an A15 Automatic Biochemistry Analyzer (Biosystems, Barcelona, Spain). Liver tissue was added to cold saline and homogenized 1 : 20 (w/v). The homogenate was centrifuged at 3000 *g* for 10 min at 4°C. The supernatant was used to detect SOD, CAT, GSH, and MDA according to the manufacturers' instructions of the kits.

### 2.6. Pathology

The fixed liver tissue was embedded in paraffin, cut into 4 *μ*m thick sections, and stained with hematoxylin and eosin.

### 2.7. Immunohistochemistry

Paraffin slices were dewaxed and then dehydrated using an ethanol gradient. Endogenous peroxidase activity was blocked with 3% hydrogen peroxide for 10 min. The tissue was microwaved with 10 mM sodium citrate buffer, and 5% bovine serum albumin was used for 30 min to block nonspecific antigens. Slices were incubated with the Nrf2 antibody (1 : 200) or phosphate-buffered saline for 12 h at 4°C. Slices were then incubated with the horseradish peroxidase-conjugated secondary antibody for 1 h at 37°C. Finally, slices were treated with the diaminobenzidine substrate-chromophore solution and stained with hematoxylin.

### 2.8. Quantitative Real-Time Polymerase Chain Reaction (qRT-PCR)

Total RNA was extracted from the liver using RNAiso Plus Reagent (Takara Biotech, Kyoto, Japan) according to the manufacturer's protocol. cDNA was produced by a reverse transcription kit (Takara Biotech). The qRT-PCR was performed using a PCR detection system (Eppendorf, Hamburg, Germany). Primers were obtained from Sangon Biological Engineering (Shanghai, China) and were shown in Table 2. Using the internal control *β*-actin, the relative mRNA levels of Nrf2, Keap1, CYP2E1, and HO-1 were calculated using the 2^−△△Ct^ method.

### 2.9. Western Blot

Liver proteins were extracted for western blotting. Fifty micrograms of protein per lane were separated by sodium dodecyl sulfate polyacrylamide gel electrophoresis and then blotted onto a polyvinylidene difluoride membrane (Bio-Rad, Hercules, CA, USA). The membrane was blocked in 5% skim milk for 3 h at room temperature and then incubated with anti-Nrf2 (1 : 1000), anti-Keap1 (1 : 1000), anti-CYP2E1 (1 : 1000), anti-HO-1 (1 : 1000), and anti-*β*-actin (1 : 1000) antibodies. After washing with tris-buffered saline/Tween 20 (TBST), membranes were incubated with an anti-rabbit IgG antibody (1 : 2000) and washed with TBST, and the proteins were detected by enhanced chemiluminescence. The intensity of each band was analyzed and quantified by ImageLab software (Bio-Rad).

### 2.10. Statistical Analysis

Data were analyzed using a one-way analysis of variance using SPSS 17.0 (IBM, Chicago, IL, USA). Dunnett's multiple comparison post hoc test was used to determine group difference. Data are expressed as means ± SD. *P* < 0.05 was considered to be statistically significant.

## 3. Results

### 3.1. Composition of QGD

**Figure 1:**
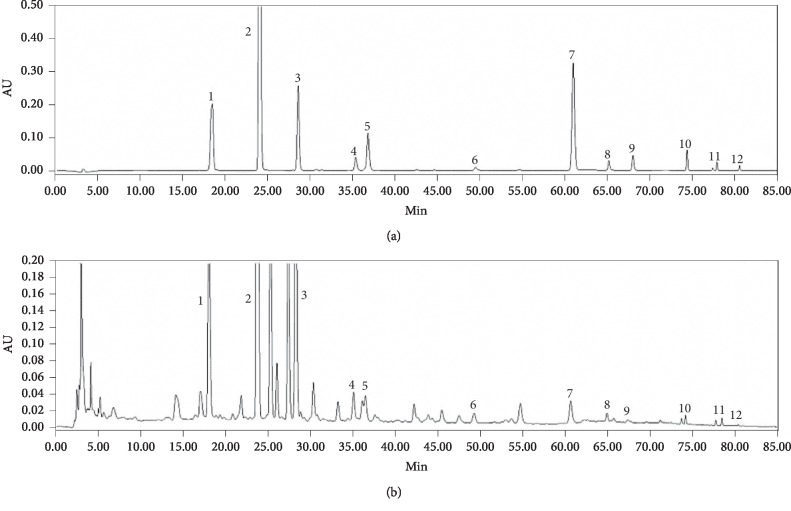
High-performance liquid chromatography reference fingerprint of the Qi-Ge decoction (QGD). Standard chemicals (a) and QGD (b) were analyzed with diode-array detection. Peak numbers indicate the following chemicals; 1: 3′-hydroxy puerarin, 2: puerarin, 3: daidzin, 4: calycosin-7-glucoside, 5: genistin, 6: hesperidin, 7: daidzein, 8: calycosin, 9: genistein, 10: formononetin, 11: nobiletin, and 12: tangeretin.

Qualitative analysis of QGD was performed by HPLC-DAD, and its chromatographic profile was compared to retention times and absorption spectra of standard chemicals. According to the HPLC spectrum, QGD contained the following compounds: 3′-hydroxy puerarin, calycosin, calycosin-7-glucoside, daidzein, daidzin, formononetin, genistin, hesperidin, genistein, puerarin, nobiletin, and angeretin (Figure 1).

### 3.2. Effects of QGD on Serum Biomarkers in Rats with CCl_4_-Induced Liver Injury

**Figure 2:**
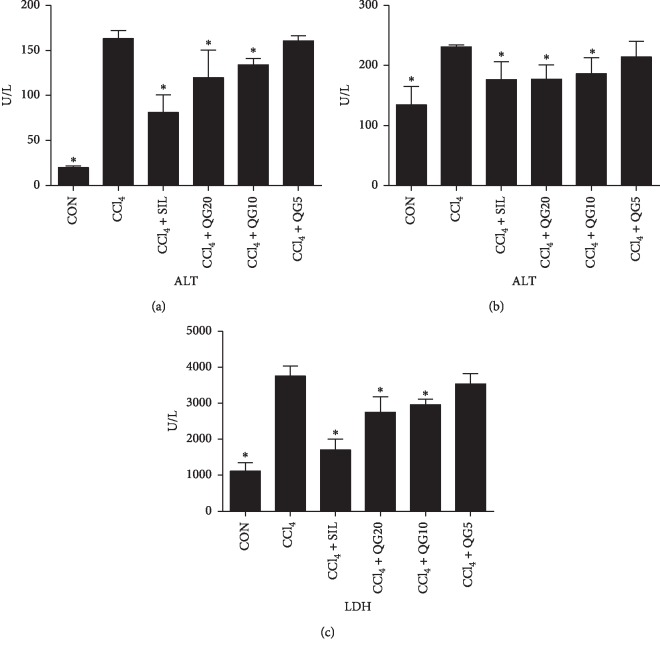
Effects of the Qi-Ge decoction (QGD) on serum (a) alanine aminotransferase (ALT), (b) aspartate aminotransferase (AST), and (c) lactate dehydrogenase (LDH) activities in rats with CCl_4_-induced liver injury. Data are expressed as means ± SD (*n*=10). ^*∗*^*P* < 0.05 compared with the CCl_4_ alone group.

Serum ALT, AST, and LDH levels were measured to assess the severity of liver injury and the hepatoprotective effects of QGD. The results (Figures 2(a)–2(c)) showed that activities of serum ALT, AST, and LDH were significantly increased 24 h after induction of liver injury by CCl_4_(*P* < 0.05). High and moderate dose of QGD significantly reduced serum ALT, AST, and LDH activities, compared with the CCl_4_ alone group (*P* < 0.05). Silymarin significantly reduced serum activities of ALT, AST, and LDH compared with the control group (*P* < 0.05).

### 3.3. Effects of QGD on Liver Antioxidant and MDA Levels after Treatment with CCl_4_

**Figure 3:**
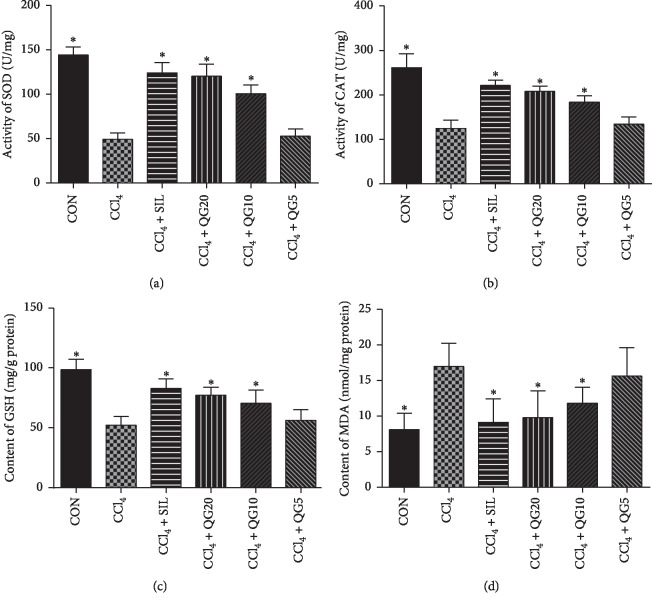
Effects of the Qi-Ge decoction (QGD) on the activities of (a) superoxide dismutase (SOD) and (b) catalase (CAT), and levels of (c) glutathione (GSH) and (d) malondialdehyde (MDA) in rats with CCl_4_-induced liver injury. Data are expressed as means ± SD (*n*=10). ^*∗*^*P* < 0.05 compared with the CCl_4_ alone group.

Compared with the control group, liver activities of SOD and CAT, and the level of GSH, were decreased significantly in CCl_4_-damaged livers (Figures 3(a)–3(c)) (*P* < 0.05). These changes were prevented by treatment with silymarin and high or moderate dose of QGD (*P* < 0.05), but not significantly affected by the low dose of QGD (*P* > 0.05).

Silymarin and high or moderate dose of QGD significantly reduced MDA (Figure 3(d)) levels due to CCl_4_-induced liver injury (*P* < 0.05), though the levels remained elevated compared to the control group (*P* < 0.05). Low dose of QGD did not reduce elevated MDA levels (*P* > 0.05).

### 3.4. Histopathological Assessment of Liver Damage

**Figure 4:**
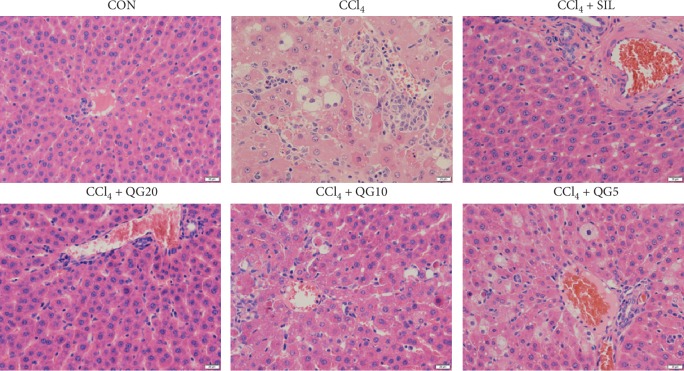
Effects of the Qi-Ge decoction (QGD) on histopathological changes in rats with CCl_4_-induced liver injury (hematoxylin and eosin staining; 400×).

Liver tissue of normal rats did not show any histopathological abnormalities; it was cord-like with no pathological changes such as edema, steatosis, or inflammatory cell infiltration. In contrast, hepatocytes in the CCl_4_-treated group were extensively steatotic and arranged irregularly. At the same time, varying sizes of fat vacuoles were seen diffusely in the cytoplasm, and some hepatocyte areas were spotted and focally necrotic with inflammatory cell infiltration and hyperplasia. Silymarin treatment significantly improved hepatocyte abnormalities caused by CCl_4_. Hepatocytes in rats treated with high and moderate doses of QGD appeared close to normal, and the above pathological changes were improved compared with the model group. In particular, CCl_4_-induced liver injury was the least severe in the high-dose QGD group, and there were almost no fat vacuoles in the visual field, and no degeneration, necrosis, or other pathological changes in hepatocytes. However, the low dose of QGD did not significantly improve the histopathological changes caused by CCl_4_ (Figure 4).

### 3.5. Effects of QGD on mRNA and Protein Expression of Nrf2, Keap1, HO-1, and CYP2E1 in Rats with CCl_4_-Induced Liver Injury

**Figure 5:**
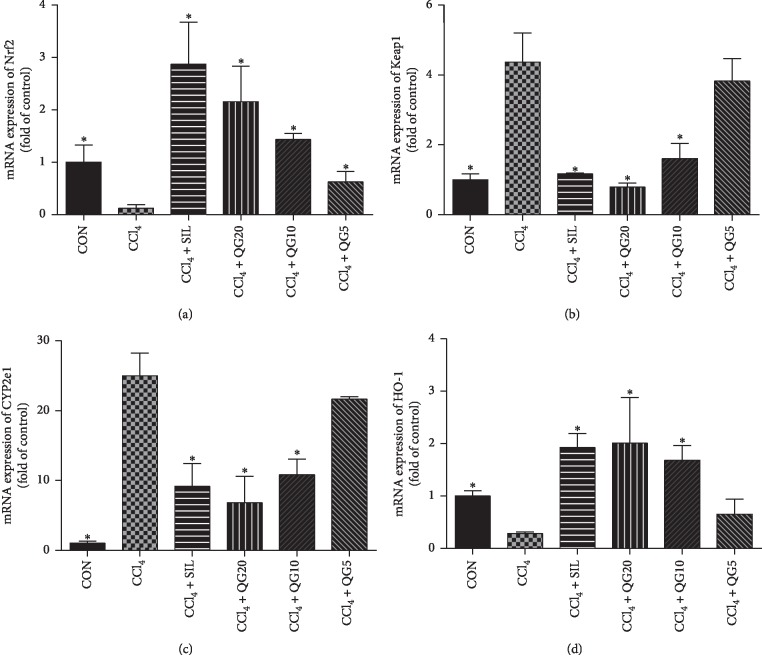
Effects of the Qi-Ge decoction (QGD) on the expression of nuclear factor erythroid 2-related factor 2 (Nrf2), Kelch-like ECH-related protein 1 (Keap1), cytochrome P450 (CYP)2E1, and heme oxygenase-1 (HO-1) mRNAs in rats with CCl_4_-induced liver injury. Data are expressed as means ± SD (*n*=3). ^*∗*^*P* < 0.05 compared with the CCl_4_ alone group.

**Figure 6:**
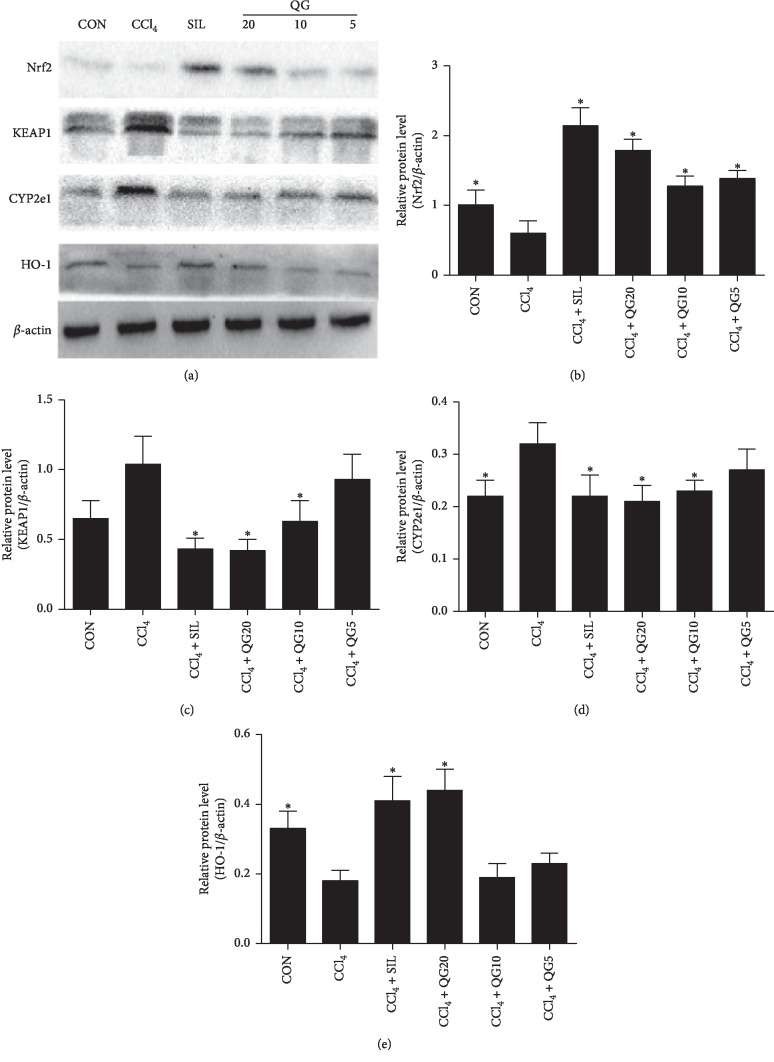
Effects of the Qi-Ge decoction (QGD) on the expression of nuclear factor erythroid 2-related factor 2 (Nrf2), Kelch-like ECH-related protein 1 (Keap1), cytochrome P450 (CYP)2E1, and heme oxygenase-1 (HO-1) proteins in rats with CCl_4_-induced liver injury. Data are expressed as means ± SD (*n*=3). ^*∗*^*P* < 0.05 compared with the CCl_4_ alone group.

**Figure 7:**
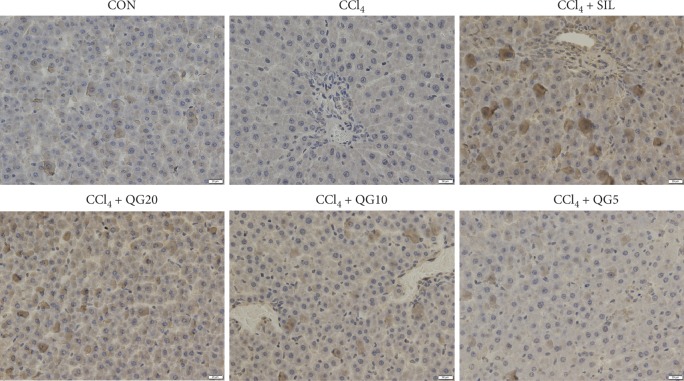
Representative immunohistochemical image of Nrf2 in rats with CCl_4_-induced liver injury (400×).

The mRNA and protein levels of Nrf2 and HO-1 were significantly elevated in the silymarin and QGD groups compared to rats treated with CCl_4_ alone. Silymarin and QGD significantly reduced the mRNA and protein levels of Keap1 and CYP2E1 compared with the model group (Figures 5 and 6). Immunohistochemistry confirmed that the administration of silymarin and QGD significantly increased Nrf2 protein expression in hepatocytes compared to rats treated with CCl_4_ alone (Figure 7).

### 3.6. QGD Protects Against Liver Injury Induced by CCl_4_ via Regulating Nrf2 Antioxidative Pathway

**Figure 8:**
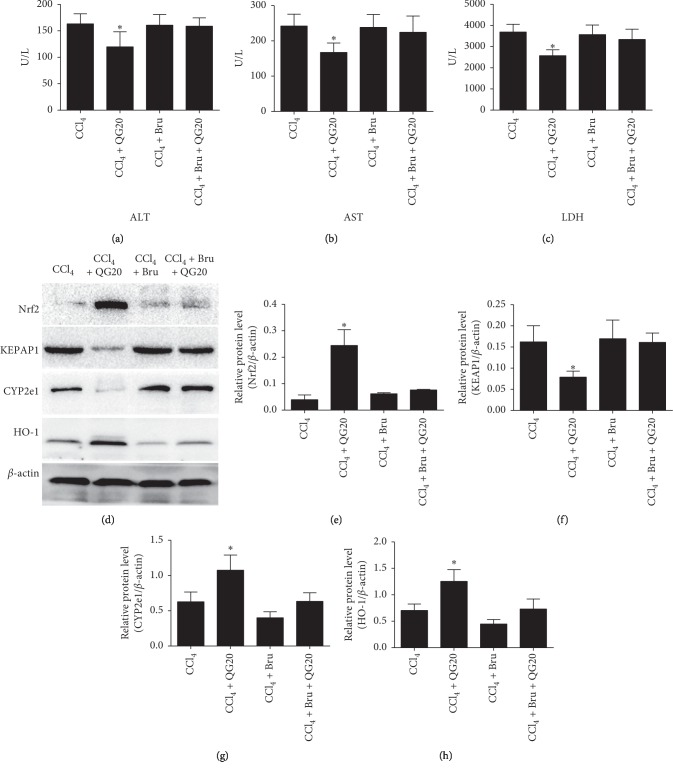
QGD protects against CCl_4_-induced liver injury via activation of the Nrf2 antioxidative pathway. Data are expressed as means ± SD (*n*=3). ^*∗*^*P* < 0.05 compared with the CCl_4_ alone group.

To explore the mechanism of QGD liver protection, we used the Nrf2 inhibitor brusatol to inhibit the relevant antioxidative pathway and measured the protein expression level. As shown in Figures 8(a)–8(c), the Nrf2 inhibitor brusatol significantly reversed the decreased activity of serum ALT, AST, and LDH induced by 20 g/kg QGD after CCl_4_-induced liver injury (*P* < 0.05). As shown in Figures 8(d)–8(h), 20 g/kg QGD significantly increased Nrf2 and HO-1 compared to the CCl_4_ group and inhibited protein expression levels of Keap1 and CYP2E1. After treatment with brusatol or brusatol + QGD, Nrf2 and HO-1 protein expression levels were inhibited, but expression of Keap1 and CYP2E1 was increased. These data indicate that QGD protects the liver from CCl_4_-induced damage by modulating the Nrf2 antioxidative pathway.

## 4. Discussion

When excessive xenobiotics, such as toxic drugs, chemicals, and viruses, cannot be eliminated in a timely manner, they can damage the liver (often by ROS), leading to acute or chronic liver injury [15–17]. CCl_4_ is a toxic xenobiotic that causes hepatocyte necrosis and liver damage and is often used as a model toxin for the induction of liver injury and evaluating the protective effect of drugs [18,19]. Under the catalysis of CYP2E1 in hepatocytes, CCl_4_ is stripped of a chlorine to form the trichloromethyl radical. This radical spontaneously loses another chlorine to form the highly oxidative metabolite carbene, which can cause hepatocyte degeneration and necrosis by inducing oxidative stress. The resulting liver injury can progress to liver fibrosis and sclerosis [20]. Serum enzymes, such as ALT, AST, and LDH, are useful markers for detecting liver damage because activities of these enzymes are markedly elevated when hepatocytes are damaged [21].

A previous study measuring ALT, AST, and LDH in serum showed that actinomycete extracts protected against CCl_4_-induced liver damage, and that this extract prevented necrosis of hepatocytes and infiltration of inflammatory cells [22]. In the current study, a significant increase in the serum activities of ALT, AST, and LDH in CCl_4_-treated rats indicated the presence of severe liver damage. Importantly, QGD reduced the serum activities of ALT, AST, and LDH, suggesting that it had a protective effect on CCl_4_-induced liver injury. Furthermore, as the dose of QGD increased, the activities of ALT, AST, and LDH gradually decreased. Histopathological results confirmed that, compared with the control group, high-dose QGD treatment reduced CCl_4_-induced hepatocyte injury, mainly manifested by hepatocytes with near-normal morphology and the almost complete absence of cell edema and steatosis. These findings further demonstrate the hepatoprotective function of QGD.

The liver maintains redox homeostasis in vivo by controlling the production and scavenging of ROS. Excess ROS are cleared by enzymes such as SOD and CAT, and both nonenzymatically and as enzyme cofactors by antioxidants such as GSH [23]. However, when the body is exposed to toxic pro-oxidant xenobiotics, the ROS level increases and the antioxidant system may be inadequate [24]. ROS interact with lipids to form MDA, which forms covalent adducts with phospholipids, DNA, and proteins, ultimately causing hepatocyte death [25].

A study has shown hepatoprotective effects of defatted extracts and flavonoids derived from A. spruneri Boiss. These compounds restored activities of the antioxidant enzymes SOD and CAT, and the level of GSH, after CCl_4_ treatment, and reduced the production of MDA both in vivo and in vitro [26]. In addition, another study has shown that ALT, AST, and LDH were reduced, and the activities of SOD and CAT, and level of GSH, were enhanced by puerarin in a CCl_4_-induced liver injury rat model [13]. These results demonstrate that the hepatoprotective effects of puerarin may be due to inhibition of lipid peroxidation and increased activities of antioxidant enzymes.

In the present study, the level of MDA was significantly reduced by treatment with silymarin or QGD in a CCl_4_-induced liver injury model in rats. In addition, the activities of SOD and CAT, and GSH level, were elevated in model rats after administration of silymarin and QGD. In general, our results suggest that inhibition of oxidative stress may be one mechanism by which QGD protects the liver from CCl_4_-induced injury. Thus, we further explored the potential antioxidant mechanisms of QGD by examining its effect on some molecular antioxidant factors.

Nrf2 acts as a transcription factor in detoxification and antioxidant processes that regulate the redox state of cells [27]. In the normal, unstressed state, Nrf2 remains at a low expression level due to targeted degradation by the proteasome under ubiquitination of Keap1. Once exposed to toxic pro-oxidant xenobiotics, such as CCl_4_, degradation of Nrf2 by the proteasome is inhibited, and it rapidly accumulates and translocates to the nucleus, initiating transcription and translation of downstream target genes, like HO-1, that promote cell detoxification and stress resistance [28–30].

Due to catalysis by CYP2E1, CCl_4_ is metabolized into highly oxidizing free radicals leading to liver damage [31–33]. To investigate whether QGD had an effect on CYP2E1 and the Keap1-Nrf2 pathway, and its downstream target genes, mRNA and protein expression of CYP2E1, Keap1, Nrf2, and HO-1 were examined in the liver. The results showed that QGD effectively increased the mRNA and protein levels of Nrf2, and the target gene HO-1, whereas the expression of Keap1 and CYP2E1 was decreased. Furthermore, immunohistochemistry showed that the accumulation of Nrf2 protein was reduced in CCl_4_-treated compared to normal rats. However, Nrf2 protein significantly accumulated in hepatocytes of the silymarin and QGD groups.

In addition, Nrf2 inhibitor brusatol was used to further confirm the mechanism of QGD in liver protection through antioxidative stress. We found that brusatol can dramatically reverse reduction of serum activity, such as ALT, AST, and LDH, caused by QGD. Furthermore, QGD-induced elevated protein expression of Nrf2 and HO-1 and decreased protein expression of Keap1 and CYP2E1 were also reversed by brusatol. Similar to the results of the current study, metabolites of curcumin, including tetrahydrocurcumin and octahydrocurcumin, greatly enhance the translation of Nrf2 to the target gene HO-1 by activating the Keap1-Nrf2 pathway and inhibiting expression of CYP2E1. This indicates that curcumin has excellent liver protective and antioxidant effects [34]. Therefore, we conclude that QGD may also show hepatoprotective effects by activating the Nrf2-Keap1 signaling pathway and inhibiting CYP2E1.

This study has a few limitations. First, the role of *Citri Reticulatae Blanco Pericarpium* in the protective function of QGD has not yet been elucidated. Second, the mechanism by which QGD activates the Nrf2 pathway was not confirmed in this study. Therefore, in subsequent studies, the potential role of *Citri Reticulatae Blanco Pericarpium* in the protective effect of the QGD formulation, as well as the effect of QGD on liver injury in Nrf2 knockout mice or mice with Nrf2 protein blockade, must be explored.

## 5. Conclusion

Overall, the results of this study show that QGD has a protective effect on CCl_4_-induced liver injury. This protection seems to be related to activation of the Nrf2 antioxidative pathway.

## Figures and Tables

**Table 1:** The related information about QGD.

Scientific name	Chinese name	Weight (g)	Ratio (%)
*Astragalus membranaceus* (Fisch)	Huangqi	30	66.70
*Puerariae Lobatae Radix*	Gegen	10	22.20
*Citrus Reticulata Blanco* (Rutaceae)	Chenpi	5	11.10

**Table 2:** Primer sequences used for the quantitative real-time polymerase chain reaction.

Gene	Forward primer (5′-3′)	Reverse primer (5′-3′)
Nrf2	AGCAACTCCAGAAGGAACAGGAGA	CTTGTTTGGGAATGTGGGCAACCT
Keap1	TGCTCAACCGCTTGCTGTATG	CCAAGTGCTTCAGCAGGTACA
CYP2E1	AGCACAACTCTGAGATATGG	ATAGTCACTGTACTTGAACT
HO-1	GGGTCCTCACACTCAGTTTC	CCAGGCATCTCCTTCCATTC
*β*-Actin	GAGACCTTCAACACCCCAGC	CACAGAGTACTTGCGCTCAG

## Data Availability

The data used to support the findings of this study are available from the corresponding author upon request.

## Abbreviations

ALT: Alanine aminotransferase;
AST: Aspartate aminotransferase;
CAT: Catalase;
CCl4: Carbon tetrachloride;
CYP2E1: Cytochrome P450 2E1;
GSH: Glutathione;
HO-1: Heme oxygenase-1;
HPLC-DAD: High-performance liquid chromatography with diode-array detection;
Keap1: Kelch-like ECH-related protein 1;
LDH: Lactate dehydrogenase;
MDA: Malondialdehyde;
Nrf2: Nuclear factor erythroid 2-related factor 2;
QGD: Qi-Ge decoction;
SOD: Superoxide dismutase.
